# "Riders" by Coroners' Juries

**Published:** 1916-04-29

**Authors:** 


### "RIDERS" BY CORONERS' JURIES.

The reform of the Coroner's Court is one of the many questions which though ripe for solution are at present undergoing the postponing influence of the war. Somewhere in the Home Office is a pigeon-hole in which reposes the Report of a Departmental Committee appointed in 1908, a date which suggests that influences other than the war must bear some responsibility for the legislative delay. We by no means suggest that this Report is in all respects satisfactory, but presumably, if and when it is made the basis of a Government Bill, there will be an opportunity to explore the whole subject. It is to be hoped that the medical profession will take steps to secure that their views are fully represented and pressed in the discussion. There are certain aspects of the question which are of great importance to the profession.

A limited view of the subject is provided by the power of the Coroner's jury to add a "rider" to their verdict. We certainly are not going to say that none of these "riders" have ever been of value, but when they reflect on the conduct of an individual there are several reasons why they should be kept within narrow bounds, even if the power to frame them is not abolished altogether. The injustice which has often been inflicted on medical practitioners by these "riders" has not infrequently been considerable, and their true character may therefore reasonably be pointed out in these columns. First, as bearing on such findings, it must be remembered that the legal rules of evidence do not hold sway in a Coroner's Court. Questions may be put and answers given which before any other legal tribunal would promptly be ruled out as hopelessly irrelevant. These questions and answers may seriously reflect on the personal or professional character of an individual, but this consideration will not give him any *locus standi*. He cannot insist upon either being called as a witness or being represented by counsel, and a personal protest may be suppressed by a threat to commit him for contempt of court. Again, whatever view be expressed by either the Coroner or the jury is a "privileged" statement, and no one who may think himself wronged by it has any legal remedy. He can neither sue for damages nor can he carry the question to any court of appeal. This last-mentioned position demands special emphasis. Put at its highest the Coroner's tribunal can hardly hope to rank higher than a Court of first instance; and yet, though either its president or its jury may register a finding which may destroy the livelihood or ruin the reputation of one of the citizens, this latter has neither redress nor remedy. We readily recognise that many Coroners appreciate the difficulties of medical practitioners concerned with questions discussed in the Coroner's Court, but not all merit this recognition, and in some instances the unfairness of a jury has rather been promoted than controlled. When legislation on the subject becomes practicable the possibility of such wrongs and of other recognised anomalies in Coroner's Court procedure must be brought to an end.

